# Elevated de novo protein synthesis in FMRP-deficient human neurons and its correction by metformin treatment

**DOI:** 10.1186/s13229-020-00350-5

**Published:** 2020-05-27

**Authors:** Kagistia Hana Utami, Nur Amirah Binte Mohammad Yusof, Jing Eugene Kwa, Ulla-Kaisa Peteri, Maija L. Castrén, Mahmoud A. Pouladi

**Affiliations:** 1 Translational Laboratory in Genetic Medicine, Agency for Science, Technology and Research, Singapore (A*STAR), 8A Biomedical Grove, Immunos, Level 5, Singapore, 138648 Singapore; 2 Department of Physiology, Faculty of Medicine, University of Helsinki, Helsinki, Finland; 3 Yong Loo Lin School of Medicine, National University of Singapore, Singapore, Singapore

**Keywords:** Fragile X syndrome, Human stem cells, Protein synthesis, Therapy

## Abstract

FXS is the most common genetic cause of intellectual (ID) and autism spectrum disorders (ASD). FXS is caused by loss of FMRP, an RNA-binding protein involved in the translational regulation of a large number of neuronal mRNAs. Absence of FMRP has been shown to lead to elevated protein synthesis and is thought to be a major cause of the synaptic plasticity and behavioural deficits in FXS. The increase in protein synthesis results in part from abnormal activation of key protein translation pathways downstream of ERK1/2 and mTOR signalling. Pharmacological and genetic interventions that attenuate hyperactivation of these pathways can normalize levels of protein synthesis and improve phenotypic outcomes in animal models of FXS. Several efforts are currently underway to trial this strategy in patients with FXS. To date, elevated global protein synthesis as a result of FMRP loss has not been validated in the context of human neurons. Here, using an isogenic human stem cell-based model, we show that de novo protein synthesis is elevated in FMRP-deficient neural cells. We further show that this increase is associated with elevated ERK1/2 and Akt signalling and can be rescued by metformin treatment. Finally, we examined the effect of normalizing protein synthesis on phenotypic abnormalities in FMRP-deficient neural cells. We find that treatment with metformin attenuates the increase in proliferation of FMRP-deficient neural progenitor cells but not the neuronal deficits in neurite outgrowth. The elevated level of protein synthesis and the normalization of neural progenitor proliferation by metformin treatment were validated in additional control and FXS patient-derived hiPSC lines. Overall, our results validate that loss of FMRP results in elevated de novo protein synthesis in human neurons and suggest that approaches targeting this abnormality are likely to be of partial therapeutic benefit in FXS.

## Introduction

Fragile X syndrome (FXS) is the leading genetic cause of intellectual (ID) and autism spectrum disorders (ASD) [1]. The disease is caused by the expansion of a CGG trinucleotide repeat tract in the promoter region of *FMR1* leading to epigenetic silencing and loss of its protein product, FMRP [2]. Individuals with FXS present with hypersensitivity, anxiety, epilepsy and cognitive problems. In addition, FXS patients exhibit characteristic physical features that include long face, prominent ears and macro-orchidism [3].

FMRP is a brain-enriched RNA-binding protein involved in the translational regulation of a large number of mRNAs that encode genes involved in neuronal development and function [4, 5]. It is localized in the somatodendritic compartment of neurons where it represses the translation of target mRNAs by stalling the ribosomes. Upon activation of translation initiation factor signals, FMRP-mediated repression is abolished to promote newly synthesized proteins that are required for a myriad of cellular and neuronal functions including synaptic plasticity [5]. Studies in *Fmr1* knockout (KO) mice have shown that absence of FMRP leads to abnormal signalling of cell-surface receptor pathways, of which metabotropic glutamate receptor 5 (mGluR5) has been the most widely studied. This in turn results in an elevation of global protein synthesis that has been observed in multiple brain regions of FXS animal models [6, 7]. mGluR receptors activate the phophoinositide-3-kinase-Akt signalling to mechanistic target of Rapamycin complex 1 (mTORC1) and/or Ras-Raf activation leading to a hyper-sensitized extracellular signal regulated kinase 1/2 (ERK1/2) pathway. The increase in global protein synthesis results in part from abnormal activation of key protein translation pathways downstream of ERK1/2 and mTORC1 signalling. Both signalling pathways converge to activate components of the eukaryotic cap-dependent translation machinery [8, 9]. Pharmacological and genetic approaches that attenuate hyperactivation of these pathways can normalize levels of protein synthesis and have been shown to improve phenotypic outcomes in animal models of FXS. These include several mGluR5 antagonists, GABA agonists, statins, lithium and ribosomal protein tyrosine kinase S6 (S6K) inhibitors [8, 10–13].

Several therapeutic strategies are currently being evaluated in clinical trials for FXS, including treatments aimed at normalizing protein synthesis. Altered protein synthesis has been previously examined directly in FXS lymphoblastoid cell lines and fibroblasts and indirectly in FXS patients using positron-emission tomography [6, 14–16]. However, assessment in human neural cells, the cell types of most relevance to neurodevelopmental and neurological manifestations of FXS, remains lacking. The aim of this study is to evaluate the protein synthesis status and the effect of its normalization on phenotypic deficits in human FMRP-deficient neural cells derived from an isogenic human stem cell-based model.

## Materials and methods

### Cell culture

The cell lines used in this study are H1/WA01 hESC (WiCell, Wisconsin) and isogenic *FMR1*KO hESC lines generated by CRISPR/Cas9 targeting exon 3 of *FMR1* gene in the H1 hESC line. Details of the generation of *FMR1*KO hESC lines are described in Utami et al., *BioRxiv* 2019 [17]. Two control (HEL11.4 and HEL23.3) and two FXS (HEL69.5 and HEL70.3) human-induced pluripotent stem cells (hiPSCs) were included for further assessment of the protein synthesis phenotypes and metformin treatment effects [18].

### Neural differentiation

hESCs were induced into neural progenitor cells (NPCs) according to a previously published protocol [19]. Briefly, single-cell dissociated hESC at a density of 30,000 cells/cm^2^ was plated in neural induction media (NIM, DMEM/F12:NeuroBasal media 1:1 with 1% N2, 2% B27, 1% PenStrep, 1% GlutaMax, 10 ng/ml hLIF and 5 μg/ml Bovine Serum Albumin) containing 4 μM CHIR99021 (Tocris), 3 μM SB431542 (Sigma) and 0.1 μM Compound E (Millipore) for the first 7 days. The culture was then split at a 1:3 ratio for the next five passages using Accutase in NIM without Compound E on Matrigel-coated plates.

For neuronal differentiation, NPCs were plated at a density of 20,000 cells/cm^2^ on 50 μg/ml poly-L-ornithine/10 μg/ml laminin-coated plates and grown in NeuroDiff media (DMEM/F12/Neurobasal media (1:1) supplemented with 1% N2, 2% B27, 20 ng/ml GDNF (R&D Systems), 20 ng/ml BDNF (R&D Systems), 300 μM dibutyryl-cyclic AMP (D0260, Sigma Aldrich) and 200 nM L-Ascorbic Acid (A4403, Sigma Aldrich)) for at least 3 weeks. Medium was changed every 2–3 days.

### Metformin treatment

NPCs or neurons were seeded on Matrigel-coated plates or poly-l-ornithine/laminin-coated plates, respectively at a confluency of 30–40%. One day after plating, metformin was added into the media at the concentration being evaluated. For the SUnSET protein synthesis assay, metformin was added during the puromycin incubation. For NPCs experiments, metformin was added to NPC cultured between passage 5 and 7, and media with metformin was refreshed daily for 3 days. In neurons, metformin treatment was given for ~ 7 days, and media with fresh metformin was changed every 2 days.

### Immunofluorescence staining

Cells were plated on ethanol-treated coverslips and fixed with 4% formaldehyde in phosphate buffer saline (PBS) for 15 min at room temperature. After washing with Tris-Buffered Saline (TBS), cells were incubated in blocking buffer (TBS containing 5% goat serum, 1% Bovine Serum Albumin and 0.1% Triton-X-100 (Sigma Aldrich)) for 45 min at room temperature. Primary antibodies were incubated with fixed cells overnight at 4 °C in blocking buffer without Triton-X-100. The following primary antibodies were used: anti-MAP2 rabbit polyclonal (AB5622, EMD Millipore), anti-TUJ1 mouse monoclonal (MAB1637, Merck-Millipore), anti-Nestin mouse monoclonal (MAB5326, Merck-Millipore), anti-rat BrdU (sc-56258, Santa Cruz) and anti-Ki67 mouse monoclonal (MAB4190, Merck-Millipore). Cells were subsequently stained with secondary antibodies conjugated to Alexa Fluor 555 or 488 (Molecular Probes, Thermo Fisher) for 1 h at room temperature in the dark and incubated with 1 μg/ml 4′,6-diamidino-2-phenylindole (DAPI, Sigma Aldrich) for 10 min. Images were captured using an FV1000 Inverted Confocal System.

### Cell proliferation assay

Approximately 70% confluent NPCs were treated with 50 μM BrdU for 6 h, followed by fixation with 4% formaldehyde for 15 min at room temperature. For antigen retrieval, coverslips were incubated serially three times in ice-cold 1 N HCl for 10 min, 2 N HCl for 10 min at room temperature, 2 N HCl for 20 min at 37 °C and lastly in 1 M borate buffer for 10 min. Immunofluorescence staining and imaging was performed as described in the “Immunofluorescence staining” section using anti-BrdU (sc56258, Santa Cruz) and anti-Ki67 (MAB4190, Millipore) antibodies. To determine the proportion of positive cells, images were captured from at least 10 randomly selected areas. Quantification for each sample/genotype was performed blinded from 3 coverslips per genotype/treatment. The ImageJ software was then used to compute the total number of cells (DAPI-stained nuclei) and the number of cells expressing the markers using the CellCounter Plugin. Percentage of BrdU and Ki67-positive cells was calculated from the ratio of BrdU/Ki67+ cells over a total number DAPI.

### De novo protein synthesis assay

De novo protein synthesis was measured using a previously described assay [20]. Cells were deprived of serum for 16 h and after 4 h of recovery in complete medium supplemented with 10% FBS, were treated with puromycin (5 μg/ml) for 30 min. Pre-treatment with the translation inhibitor cycloheximide (50 μM) for 15 min was used to as a control to confirm specificity of the assay. Following puromycin treatment, cells were incubated with fresh medium for 15 min then washed with ice-cold PBS and lysed directly in RIPA buffer, supplemented with cOmplete Protease Inhibitor (Roche). Samples were analysed by immunoblot, and puromycin incorporation was detected using the mouse monoclonal antibody PMY-2A4 (DSHB). Calnexin was used as loading control.

### Immunoblotting

Cells were lysed with RIPA buffer (Sigma Aldrich) containing cOmplete Protease Inhibitor cocktail tablets (Roche). Protein concentration was measured using the Bradford assay (BioRad). The samples were denatured at 70 °C for 10 min in 4× NuPAGE sample buffer and 10× NuPAGE reducing agent (Thermo Fisher). A total of 30 μg of protein per sample was separated on 12% Acrylamide FastCast kit (Bio-Rad) at 100 V for 3 h followed by transfer to nitrocellulose membrane at 120 V for 1.5 h at room temperature. The following primary antibodies were used for detection: anti-FMRP (MAB2160, Millipore; 6B8, Biolegend), anti-puromycin (DSHB, PMY2A4) and anti-Calnexin (Sigma, C7431). Alexa-Fluor 680 goat anti-mouse (Thermo Fisher) and DyLight 800 goat anti-rabbit (Rockland) were used as secondary antibodies. Membranes were imaged using the Li-Cor Odyssey infrared imaging system.

### Neurite outgrowth measurements

To assess neurite outgrowth, NPCs were plated at a density of 15,000 cells/cm^2^ in NeuroDiff media with modifications (substituting N2 with CultureOne supplement (Thermo Fisher)). The plate was imaged using an IncuCyte Zoom Imaging system (Essen Bioscience, Ann Arbor, MI) every 2 h for 7 days. Live-capture measurements were performed in 9 image field per well, *n* = 4 per genotype per treatment condition. For metformin treatment, metformin was added at day 1 post plating, and media with/without metformin was changed every 2–3 days. Cells were imaged under phase contrast, and analysis was performed using IncuCyte’s NeuroTrack module. The neurite-calling algorithm automatically traces the neurites from each cell body and masks the neurites for the entire image field, with no pre-selection of evaluated neurons. The growth rate of neurites in each well was obtained by measuring the surface area covered by neurites and expressed as mm/mm^2^.

### Statistical analysis

All statistical analysis was carried out in GraphPad Prism v7 (La Jolla, CA, USA). Statistical significance was ascertained by one- or two-way ANOVA with appropriate post hoc testing or by unpaired Student’s *t* test. Differences were considered statistically significant when *p* < 0.05.

## Results

### Transcriptome profiling analysis reveals dysregulation of protein synthesis-related pathways in FMRP-deficient neural cells

**Fig. 1:**
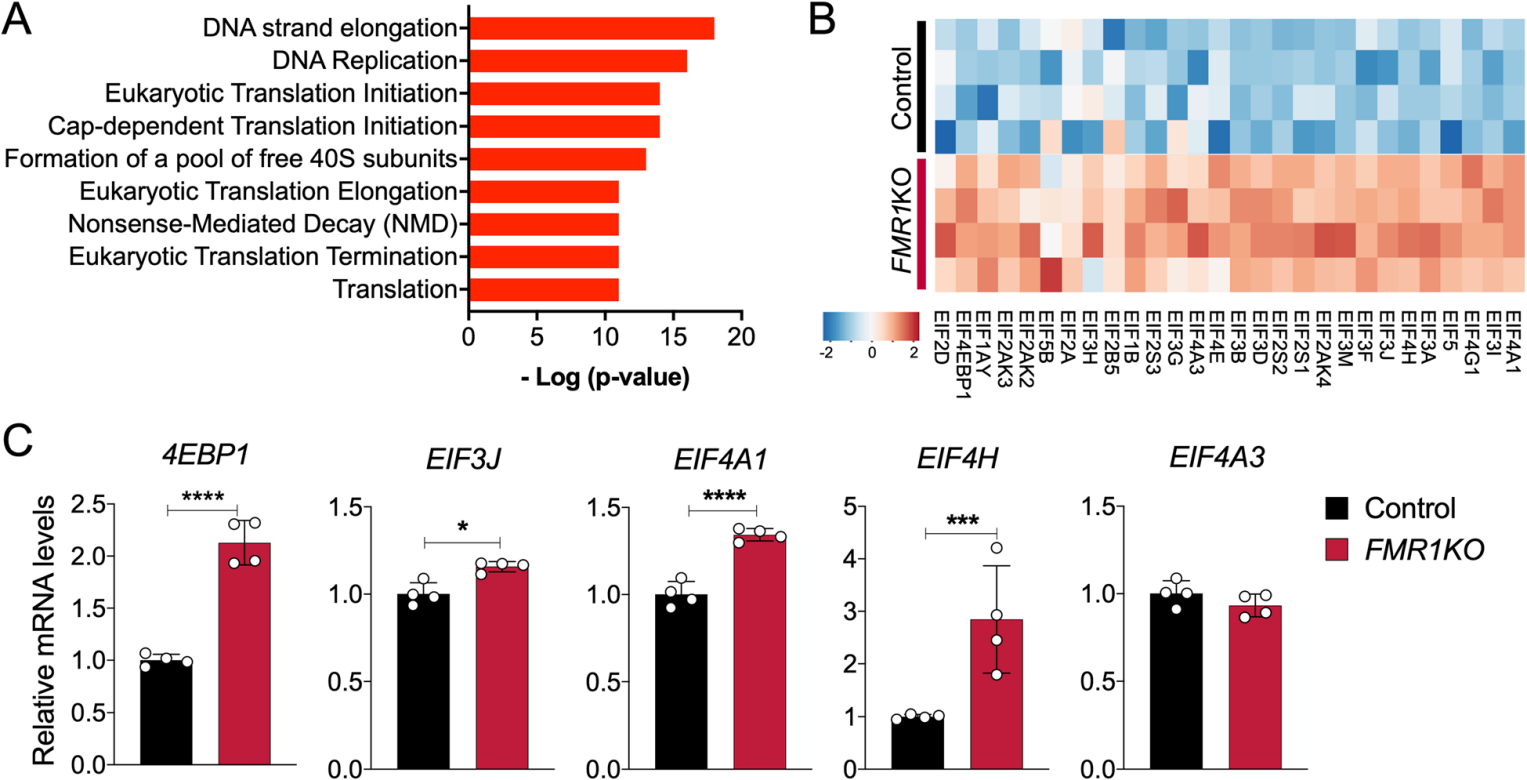
Transcriptome profiling analysis reveals dysregulation of protein translation-related pathways in FMRP-deficient neurons. **a** Functional annotation of transcripts differentially expressed in *FMR1*KO neurons shows enrichment of protein-related synthesis pathways. **b** Heatmap of representative differentially expressed genes related to protein translation in neurons. **c** Quantitative RT-PCR analysis for validation of changes for a subset of EIF-related genes in control and *FMR1*KO neurons

We previously reported transcriptome-wide profiling in *FMR1* knockout isogenic neurons derived from H1 human embryonic stem cells (hESCs) to identify key molecular signatures associated with neurodevelopmental deficits in FXS [17]. By using a focused meta-analysis of a neuronal transcriptome dataset, we found that many of the gene ontology (GO) categories associated with genes upregulated in FMRP-deficient neurons are significantly enriched in protein synthesis-related GO terms (Fig. 1a). Examinations of the genes associated within these categories, eIF-related genes, including *eIF4A1*, *eIF4E*, *eIF4G1*, *eIF1B* and *eIF2A*, were shown to be significantly upregulated in the *FMR1*KO neurons (Fig. 1b). We validated changes in four out of five EIF-related genes, namely *4EBP1*, *EIF3J*, *EIF4A1* and *EIF4H,* in the *FMR1*KO neurons by quantitative RT-PCR (Fig. 1c). These data suggest that absence of FMRP may perturb translation initiation processes in human neurons, consistent with prior studies showing that FMRP regulates initiation of translation by interacting with cap-binding translation factor eIF4E and cytoplasmic FMRP-interacting protein 1 (CYFIP1) [21].

### De novo protein synthesis is elevated in FMRP-deficient neural cells

**Fig. 2:**
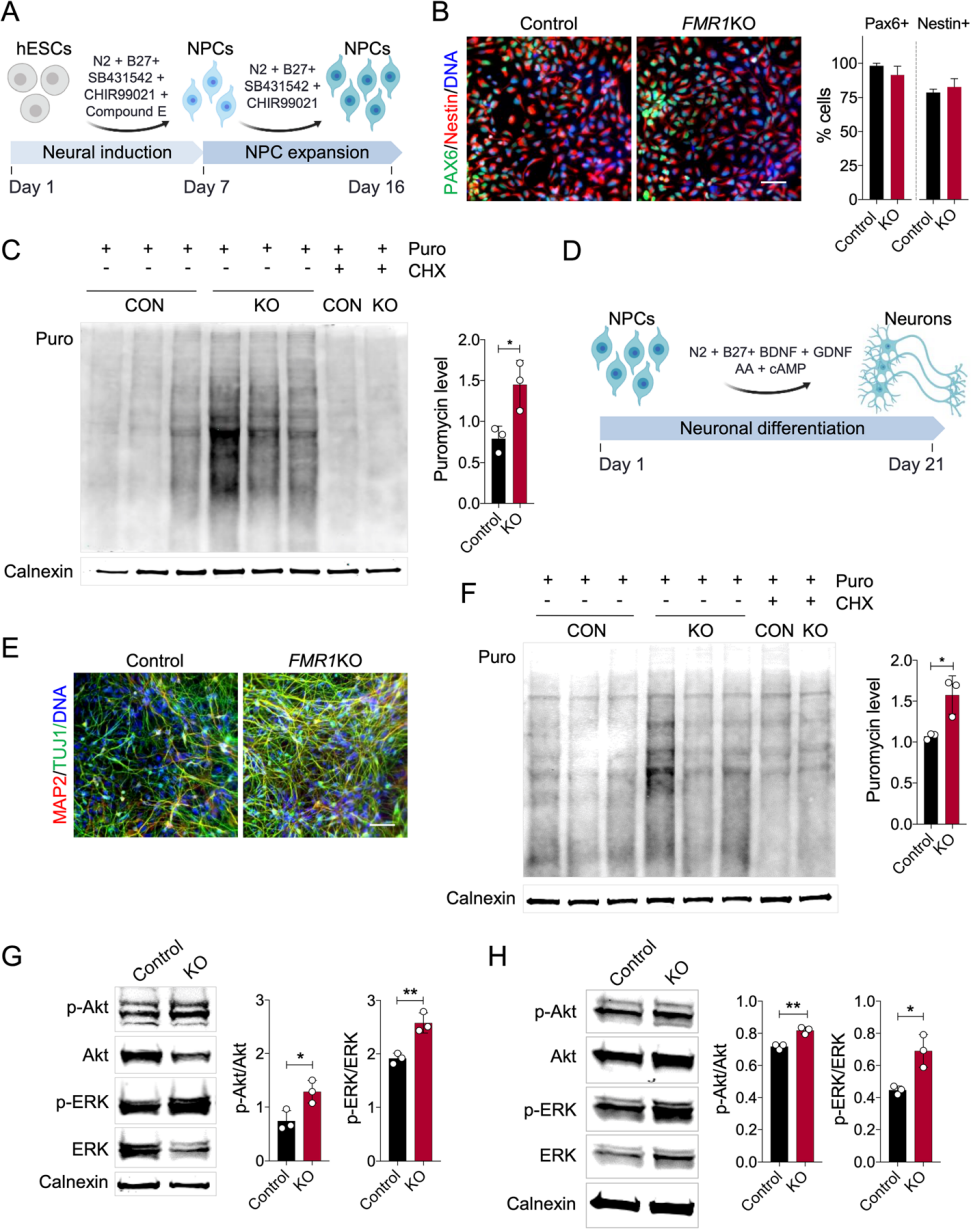
Elevated protein synthesis, p-Akt and p-ERK levels in FMRP-deficient neural progenitors and neurons. **a** Schematic overview of differentiation protocol to derive homogeneous population of NPCs using rapid neural induction protocol. **b** Control and FMR1KO hESC-derived NPCs showing comparable level of NPCs markers expression, Nestin and Pax6. The number of positive cells for PAX6 and NESTIN was quantified over the total number of cells (stained with DAPI). Scale bar = 25 μm. **c** Basal protein synthesis was determined in control and *FMR1*KO NPCs using the SUnSET assay, showing significant elevation of protein synthesis in *FMR1*KO NPCs compared to control (CON). Values shown as mean ± SEM based on *n* = 3 replicates per genotype; *p < 0.05 compared with control was determined by two-tailed unpaired Student’s *t*-test. Puromycin labelling was performed in NPCs for 30 min, after which equal amounts of protein were loaded on the gel for immunoblot analysis with anti-puromycin antibody. As a control, cycloheximide (CHX) was added to the cells and incubated for 15 min prior to Puro labelling. **d** Schematic overview of differentiation protocol to differentiate NPCs into neurons. **e** Control and FMR1KO neurons express post-mitotic neuronal markers, MAP2 and TUJ1. Scale bar = 25 μm. **f** Basal protein synthesis was assayed in neurons by SUnSET method, showing significantly elevated level of puromycin expression in *FMR1*KO compared to the control. Values shown as mean ± SEM based on *n* = 3 replicates per genotype; *p < 0.05 compared with control was determined by two-tailed unpaired Student’s *t*-test. **g** The levels of phosphorylated Akt, total Akt, phosphorylated ERK and total ERK in NPCs were determined by immunoblot. Protein loading was determined by the level of Calnexin. Representative immunoblot was shown for each sample. Quantification of protein level was performed by ImageJ. Values shown as mean ± SEM based on *n* = 3 replicates per genotype; *p < 0.05, **p < 0.01 compared with control was determined by unpaired Student’s *t*-test. **h** The levels of phosphorylated Akt, total Akt, phosphorylated ERK and total ERK in neurons were determined by immunoblot. Protein loading was determined by the level of Calnexin. Representative immunoblot was shown for each sample. Quantification of protein level was performed by ImageJ. Values shown as mean ± SEM based on *n* = 3 replicates per genotype; *p < 0.05, **p < 0.01 compared with control was determined by unpaired Student’s *t*-test

To examine the basal rate of de novo protein synthesis in FMRP-deficient neural cells, we differentiated isogenic *FMR1*KO hESCs that were derived by CRISPR/Cas9-mediated disruption of *FMR1* in H1 hESCs into neural progenitor cells (NPCs) using a previously described protocol [19] (Fig. 2a). *FMR1*KO NPCs expressed a similar level of the NPC markers Nestin and Pax6 as the isogenic control NPCs, as shown by immunofluorescence and quantification of PAX6+ and NESTIN+ cells (Fig. 2b). We next performed the SUnSET assay of global protein synthesis based on puromycin incorporation, an efficient and reliable method to measure relative rates of de novo protein synthesis [20]. We observed a significant increase in the total level of de novo protein synthesis based on elevated puromycin incorporation in the FMRP-deficient lines compared to control (Fig. 2c). There were no obvious differences in puromycin levels between the cell lines following treatment with cycloheximide (CHX), a potent blocker of mRNA translation, confirming that the puromycin measurements accurately reflect global levels of de novo protein synthesis.

To test whether FMRP-deficient neurons also exhibit this elevation in global protein synthesis, we differentiated NPCs into neurons as previously described [22] (Fig. 2d). The cells differentiated from the *FMR1*KO, and isogenic control lines expressed the neuronal markers MAP2 and TUJ1 (Fig. 2e). Analysis of protein synthesis using the SUnSET assay showed significantly elevated de novo protein synthesis in FMRP-deficient neurons compared to control neurons (Fig. 2f). Absence of FMRP in *FMR1*KO cells in NPCs and neurons was confirmed by immunoblotting (Fig. S1).

### Increased Akt and ERK1/2 activation in FMRP-deficient neural cells

Previous studies using *Fmr1*KO mice have shown that increased global protein synthesis is associated with activation of the MAPK/ERK1/2 and PI3K-Akt-mTOR pathways [8, 9]. These pathways are associated with the initiation of the 5′ cap-dependent translation of mRNAs. ERK1/2 activates the MAPK-interacting kinase (Mnk), thereby phosphorylating the eukaryotic initiation factor 4E (eIF4E) [21]. mTOR is activated by Akt, leading to phosphorylation of eIF4E binding proteins (4EBPs), which activates Eif4E. mTOR also initiate translation of 5′ TOP mRNA, which is linked to the activation of ribosomal protein S6 kinases (p70S6K) [9, 12]. To determine whether parts of these pathways were affected, we examined the phosphorylation states of ERK and Akt. We observed significant elevation in both phosphorylated Akt and ERK level in the *FMR1*KO NPCs (Fig. 2g) and neurons (Fig. 2h).

### Metformin treatment normalizes protein synthesis in FMRP-deficient neural cells in an ERK1/2- and Akt-independent manner

**Fig. 3:**
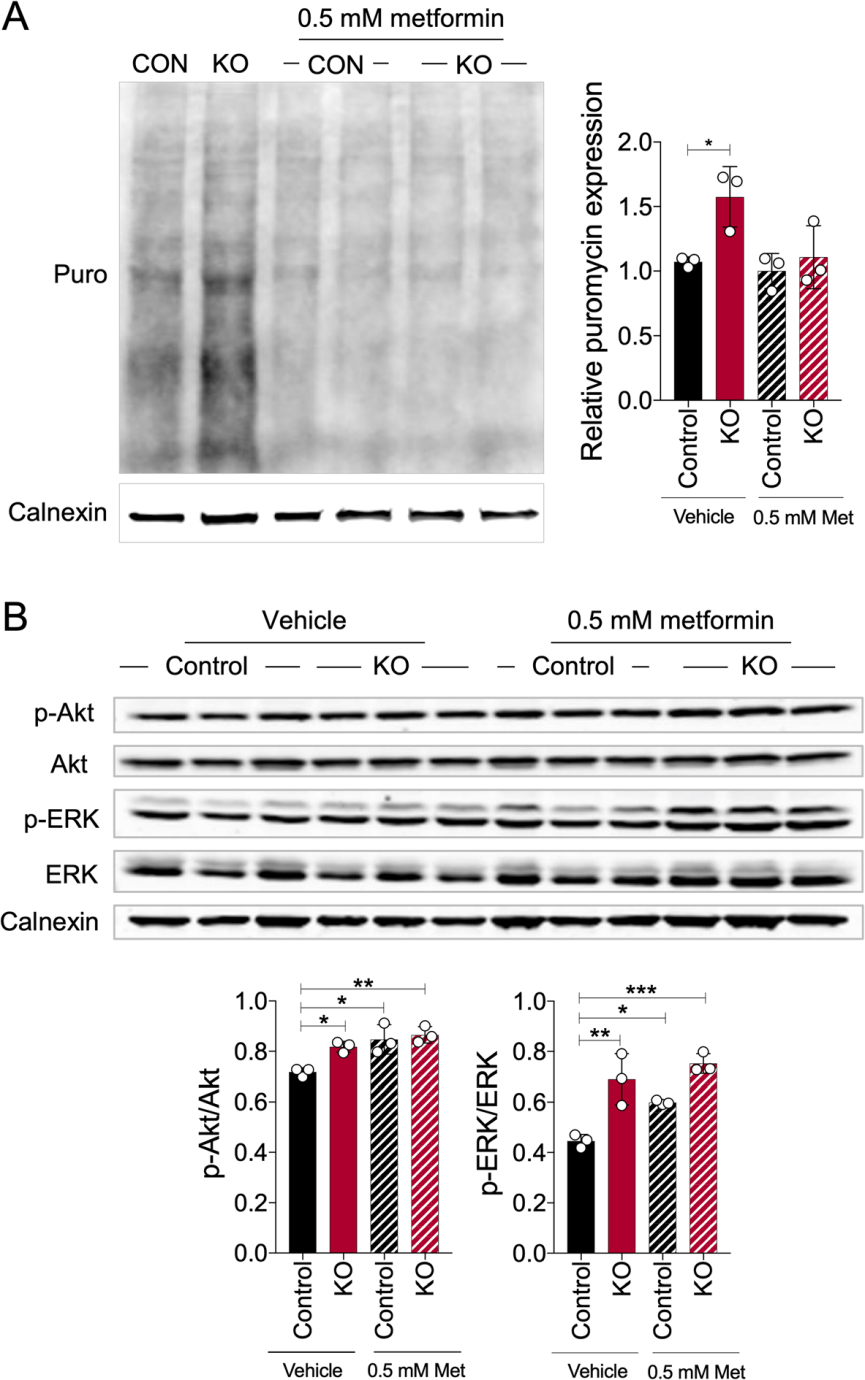
Metformin corrects the aberrantly elevated protein synthesis in FMRP-deficient neurons but does not normalize the phosphorylated levels of Akt and ERK. **a** Global protein synthesis was determined by SUnSET assay, and representative immunoblot image was shown. Metformin (0.5 mM) was added to the neurons for 72 h followed by puromycin incorporation for 30 min. Protein loading was determined by the level of Calnexin. Representative immunoblot image was shown, and relative puromycin expression was quantified by ImageJ. Values shown as mean ± SEM from three biological replicates per sample. Statistical significance was shown as *p < 0.05, **p < 0.01, as determined by one-way ANOVA. **b** Expressions of phosphorylated Akt, total Akt, phosphorylated ERK and total ERK for untreated and metformin-treated condition were assessed by immunoblot. Protein loading was determined by the level of Calnexin. Phosphorylated Akt/total Akt and phosphorylated ERK/total ERK were quantified by ImageJ. Values shown as mean ± SEM from three biological replicates per sample. Statistical significance was shown as *p < 0/05, **p < 0.01, as determined by one-way ANOVA

Metformin, a first-line therapy for type 2 diabetes, has recently been shown to normalize protein synthesis and rescue core phenotypes of *Fmr1*KO mice [23]. To test whether metformin treatment would alleviate the elevated protein synthesis in human FMRP-deficient neural cells, we treated *FMR1*KO neurons with metformin for 72 h and assessed the level of global protein synthesis using the SUnSET assay. We found that treatment with 0.5 mM of metformin, a dose previously used in dermal fibroblast cells and hepatocytes [24, 25], normalized the elevated protein synthesis in *FMR1*KO neurons (Fig. 3a). In addition, we tested the phosphorylation states of Akt and ERK1/2 and found that they were not normalized in the metformin-treated FMRP-deficient neurons (Fig. 3b). Similar results were obtained in NPCs where treatment with 0.5 mM of metformin normalized the elevation in protein synthesis but not the phosphorylation states of Akt or ERK1/2 (Figure S2A-B). We further tested two additional doses of metformin in NPCs (0.1 and 1 mM) and found that the higher (1 mM), but nor lower (0.1 mM) dose, normalized global protein synthesis but did not affect phosphorylated Akt/ERK1/2 levels in FMR1KO NPCs (Figure S3A,B). These results suggest that metformin’s effects on protein synthesis are, at least at the doses used here, independent of Akt/ERK1/2 pathway modulation.

### Normalization of protein synthesis ameliorates the increased proliferation in FMRP-deficient neural cells

**Fig. 4:**
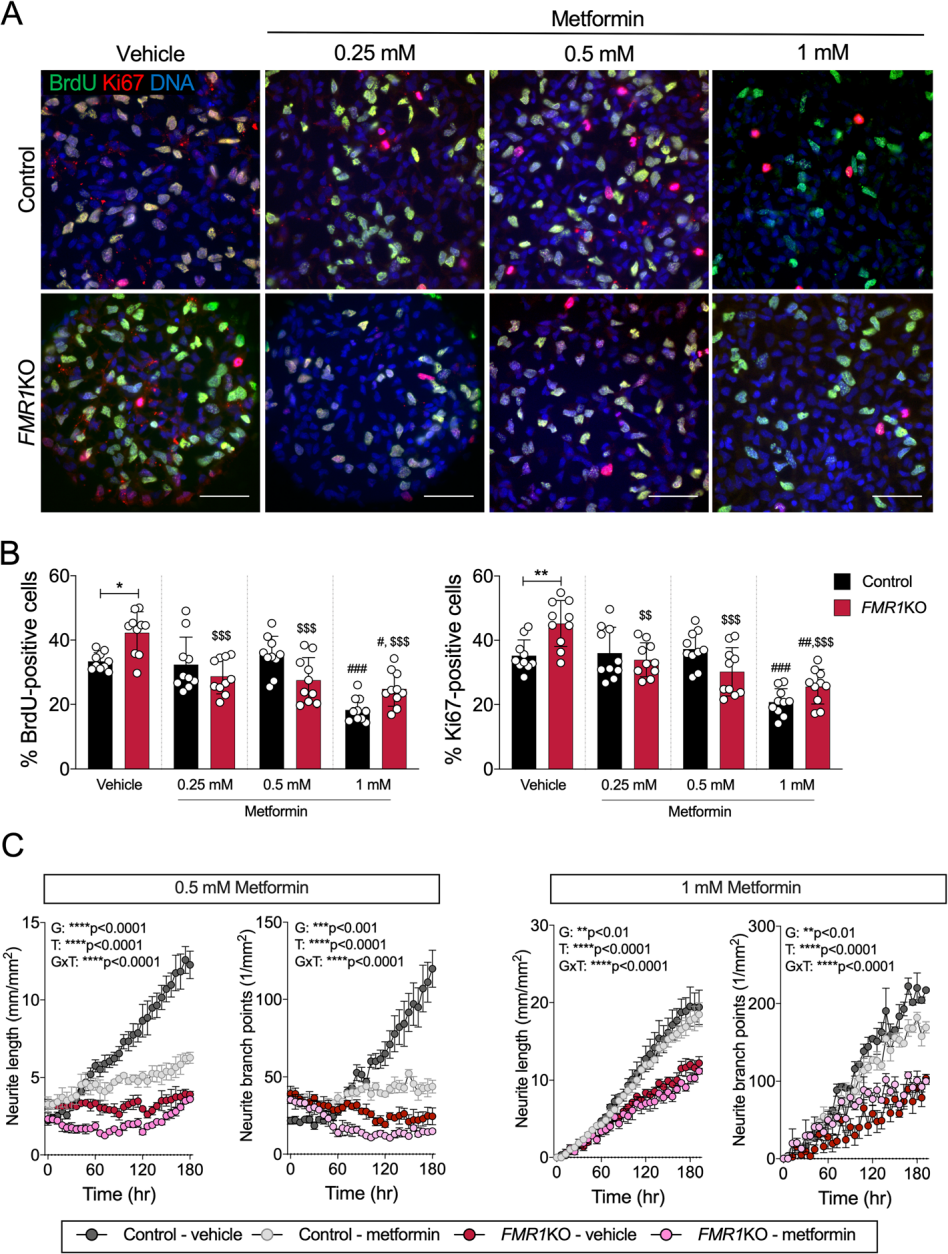
Metformin normalizes the proliferation status of FMRP-deficient NPCs but does not improve the neurite deficit in FMRP-deficient neurons. **a** Immunostaining shows proliferative markers BrdU (green) and Ki67 (red) expression. BrdU-labelling and Ki67 reveal increased proliferation in *FMR1*KO compared to control in the vehicle-treated condition. Treatment with a range of doses of metformin (0.25, 0.5 and 1 mM) ameliorates the excessive proliferation rate in the *FMR1*KO NPCs. Scale bar = 50 μm. **b** Quantification of BrdU- and Ki67-positive cells by ImageJ. Values shown as mean ± SEM based on blinded counting of 10 images per genotype per treatment condition. Statistical significance was determined by one-way ANOVA with Fisher’s LSD post hoc test; *p < 0.05, **p < 0.01 and ***p < 0.001; number sign (#) for comparison to control-vehicle and dollar sign ($) for comparison to KO-vehicle. **c** Neurite outgrowth and branching measurements for neurons treated with 0.5 or 1 mM metformin over the course of 7 days. Values shown as mean ± SD from 4 independent wells of 9 image fields per genotype

Increased proliferation has been observed in multiple FXS iPSC- and hESC-derived neural lines [26–28]. To test whether metformin treatment and the associated normalization of elevated protein synthesis can rescue phenotypic abnormalities caused by FMRP deficiency, we examined the proliferation status of FMRP-deficient NPCs. We treated NPCs with a range of doses of metformin (0.25, 0.5 and 1 mM) for 3 days and evaluated the number NPCs immunopositive for Ki67 and BrdU, two markers of cell proliferation (Fig. 4a). While the vehicle-treated *FMR1*KO NPCs were significantly more proliferative than control NPCs, the elevated proliferation rate in the *FMR1*KO NPCs was significantly rescued upon metformin treatment for all doses (Fig. 4b). However, we noted that the number of proliferating cells in the highest dose (1 mM) was significantly reduced for both control and *FMR1*KO cells, which is consistent with the known properties of metformin and its effect on proliferation of various type of cells [29–31]. Metformin treatment had no significant effect on the total number control or *FMR1*KO NPCs for duration tests (Fig. S4).

### Validation of excessive protein synthesis and metformin treatment effect in additional FXS hiPSC lines

To evaluate whether the excessive protein synthesis phenotype observed in the isogenic *FMR1*KO and control cells can be observed in patient derived cells, we performed the SUnSET assay in two additional control and FXS hiPSC-derived NPCs. We indeed observed elevated level of puromycin incorporation in FXS hiPSC-derived NPCs compared to controls (Fig. S5A). In addition, we observed a significantly increased proliferation rate in the FXS hiPSC-derived NPCs as measured by Ki67- and BrdU-positive cells (Fig. S5B,C). Similar to the isogenic *FMR1*KO lines, treatment with 0.5 mM of metformin normalized the proliferation rate of FXS hiPSC-derived NPCs to control levels (Fig. S5C).

### Normalization of protein synthesis does not improve neurite outgrowth defects in FMRP-deficient neurons

Neurite outgrowth has been shown to be affected in FXS hiPSC- and hESCs-derived neurons [32–34]. We have shown previously by using longitudinal neurite tracing that *FMR1*KO neurons have reduced neurite outgrowth compared to the control [17]. To examine whether restoration of protein synthesis corrects this neurodevelopmental abnormalities, we treated FMRP-deficient neurons with 0.5 mM and 1 mM metformin and assessed neurite outgrowth (Fig. 4c). Metformin treatment did not rescue the neurite outgrowth and branching deficits suggesting that alleviation of excessive protein synthesis may not be sufficient to correct all FMRP deficiency-related abnormalities such as neurite outgrowth deficits.

## Discussion

Effective therapies that address the core symptoms of FXS remain lacking. Therapeutic efforts to date have largely focused on normalizing synaptic plasticity deficits in FXS mouse models by targeting key receptors that regulate FMRP signalling [10, 11, 13, 15, 35]. While drugs manipulating these major receptors such as mGluR5 have shown very promising results in animal models, they have failed to meet their primary endpoints in clinical trials [36–39]. Though the reasons for this apparent lack of translatability of therapeutic success from animal models to the clinic are a subject of debate, the disappointing outcome has highlighted the need and the added-value of validating candidate therapies in the context of human physiology. In this study, we demonstrate that global protein synthesis, a current target of therapeutic efforts, is elevated in FMRP-deficient human neural cells. We further demonstrate that metformin, a candidate therapy presently in clinical trials, normalizes elevated protein synthesis and rescues some, but not all, neurodevelopmental abnormalities caused by FMRP deficiency in human neural cells.

Analysis of protein synthesis in human cells has thus far been limited to lymphoblastoid and fibroblast cell lines with variable results. For example, a recent study that examined protein synthesis in a number of FXS patient-derived cell lines found that only a subset of these lines showed elevation in protein synthesis [14]. A further examination of *Fmr1*KO mouse embryonic fibroblasts (MEFs) and neurons revealed that while there was an overall increase in protein synthesis, the correlation between matched MEFs and neurons was highly variable, leading to the conclusion that the level of protein synthesis in patient’s fibroblasts may not directly reflect the protein synthesis in neurons. These results highlighted the need for direct confirmation of protein synthesis elevation in human FMRP-deficient neurons. In our study, we showed for the first time that elevated protein synthesis occurs in both human FMRP-deficient neural progenitors and neurons.

Mechanistic studies in animal models have identified a number of pathways contributing to elevated protein synthesis in FXS. We evaluated the activation of two of these pathways, namely the Akt/mTOR and MAPK/ERK1/2 pathways. Variable results have been reported with respect to their activation at baseline. Osterweil and colleagues reported no increase in either ERK1/2 or Akt activation in *Fmr1*KO brain slices in both basal and DHPG-induced long-term depression (LTD), despite a robust increase in protein synthesis [8, 21]. In contrast, a study by Sharma and colleagues found increased Akt-mTOR pathway activation in the *Fmr1* KO hippocampus, caused by elevated expression of the PI3K enhancer protein PIKE [9]. In addition, increased level of phosphorylated ERK1/2 has been reported in the FXS human post mortem brain [40]. In our study, we observed that in both FXS isogenic hESC-derived NPCs and neurons, phosphorylated Akt and ERK levels were aberrantly elevated in parallel to excessive protein synthesis.

The ability to modulate protein synthesis pathways along with a well-established safety and tolerability profile have led to interest in metformin as a treatment for FXS. Treatment with metformin was found to rescue a number of FXS-associated deficits in *Drosophila* and mouse models of FXS [23, 41]. Chronic and acute treatment of metformin ameliorated olfactory learning and long-term memory deficits in the *dfmr1* mutants [41]. In *Fmr1*KO, mice treatment with metformin for 10 days rescued excessive translation, corrected various behavioural deficits, and decreased audiogenic seizures [23]. Mechanistically, metformin was found to correct elevated ERK, but not mTOR/Akt, signalling in *Fmr1*KO mice [23]. These findings in FXS animal models formed the basis for clinical trial of metformin as a treatment for FXS. Preliminary reports from the early stages of clinical trial in seven patients (6 adults and 1 child) showed promising improvements in speech, irritability, social behaviour and hyperactivity [42]. More recently, a report from two adult patients that have been on metformin for 1 year described significant improvements in cognitive and behaviours, as well as normalization of weight and eating habits [43].

In this study, we show that metformin does indeed normalize elevated protein synthesis in human FMRP-deficient NPCs and neurons. We further demonstrate that the improvements in neurodevelopmental abnormalities achieved were only partially, suggesting that excessive translation may not directly contribute to all FXS-associated deficits and that metformin treatment is likely to yield partial benefit.

In contrast to previous studies [23], the effect of metformin on protein synthesis was not dependent on normalization of pERK1/2 levels. Metformin is known to act through a number of pathways that converge on protein synthesis, such as the AMPK-dependent mTOR pathway and the AMPK-independent Raf/MEK/ERK signalling pathways [44]. It has been shown that the nature of the specific pathways activated by metformin depends on the tissue/cell-type, dose, as well as the treatment duration. Differences in one or more such parameters likely account for our observations of rescued protein synthesis in FMRP-deficient neural cells following metformin treatment despite the lack of effect on pERK1/2 levels.

## Limitations

The limitations of the study include the need to delineate in more detail the mechanism(s) by which metformin normalizes protein synthesis in FMRP-deficient human neurons, the assessment of the effect of metformin on a broader range of phenotypic abnormalities, as well as the concurrent assessment of the effect of metformin on protein synthesis and phenotypic deficits in the same cell population.

## Conclusion

In this study, we show that FMRP deficiency results in increased de novo protein synthesis in human neural cells as well as elevated ERK1/2 and Akt signalling. We further show that treatment with metformin normalizes in an Akt/ERK1/2-independent manner the elevation in protein synthesis and rescues some, but not all, neurodevelopmental abnormalities in FMRP-deficient neural cells. Overall, our study validates metformin as a modulator of protein synthesis in human neurons, suggests that its therapeutic effects are likely to be partial, and supports its further clinical development.

## Supplementary information

**Additional file 1: Figure S1.** Assessment of FMRP expression in control and *FMR1*KO ESC, NPC and neurons. The expression of FMRP was assessed in ESC, NPCs and neurons by immunoblot. *FMR1*KO showed absence of FMRP expression in all cell types. CON = control; KO = *FMR1*KO. **Figure S2.** Assessment of *de novo* protein synthesis and phosphorylation of ERK1/2 and Akt in FXS NPCs treated with 0.5 mM metformin. (A) Protein synthesis (SUnSET assay) was performed in NPCs treated with 0.5 mM metformin. A representative immunoblot image is shown with 2 replicates per group. Relative expression of puromycin was quantified by ImageJ and normalized to control-vehicle. Values shown as mean ± SEM (n=4 per group). *p < 0.05 and **p < 0.01 by one-way ANOVA with Fisher’s LSD post-hoc test. (B) Levels of phosphorylated Akt, total Akt, phosphorylated ERK and total ERK in untreated and metformin-treated NPCs were assessed by immunoblot. Values shown as mean ± SEM (n=3 per group). *p < 0.05 by one-way ANOVA with Fisher’s LSD post-hoc test. **Figure S3.** Assessment of *de novo* protein synthesis and phosphorylation of ERK1/2 and Akt in FXS NPCs treated with 0.1 and 1 mM metformin (A) Protein synthesis (SUnSET assay) was performed in NPCs treated with 0.1 and 1 mM metformin. A representative immunoblot image is shown. Relative expression of puromycin was quantified by ImageJ and normalized to the control-vehicle. Values shown as mean ± SEM from three replicates per genotype, from two immunoblot experiments. * p < 0.05 as determined by one-way ANOVA with Tukey post-hoc test. (B) Expression of phosphorylated Akt, total Akt, phosphorylated ERK and total ERK for untreated and metformin-treated condition was assessed by immunoblotting. A representative immunoblot image is shown. Values shown as mean ± SEM (n=4 per group). *p < 0.05, as determined by two-way ANOVA with Fisher’s LSD post-hoc test. **Figure S4.** No effect of metformin treatment on control or *FMR1*KO NPC counts. Total number of control and *FMR1*KO cells (based on DAPI-positive cells) in NPCs cultures treated with vehicle or metformin (1 mM). There were no significant differences among the groups and treatment conditions. Values shown are based on 10 image fields per group and treatment condition. **Figure S5.** Assessment of *de novo* protein synthesis and metformin effect on proliferation in FXS and control hiPSC-derived NPCs. (a) Protein synthesis (SUnSET assay) was performed in 2 Control and 2 FXS hiPSC-derived NPCs. Relative expression of puromycin was quantified by ImageJ. Values shown as mean ± SEM from three replicates per genotype. *p < 0.05 and **p < 0.01 by one-way ANOVA with Fisher LSD post-hoc test; (b) Immunostaining shows proliferative markers BrdU (Green) and Ki67 (Red) expression. BrdU labelling and Ki67 reveals increased proliferation in FXS iPSC-derived NPCs compared to control in the vehicle-treated condition. Treatment with 0.5 mM metformin ameliorates the excessive proliferation rate in the FXS hiPSC-derived NPCs. Scale bar = 50 μm; (c) Quantification of BrdU- and Ki67-positive cells by ImageJ. Values shown as mean ± SEM based on blinded counting of 8 images from three coverslips per cell line.

## Data Availability

Not applicable.

## References

[1] Hagerman RJ, et al. Fragile X syndrome. Nat Rev. Disease primers. 2017. 10.1038/nrdp.2017.65. 10.1038/nrdp.2017.65 28960184

[2] Verkerk Annemieke J.M.H., Pieretti Maura, Sutcliffe James S., Fu Ying-Hui, Kuhl Derek P.A., Pizzuti Antonio, Reiner Orly, Richards Stephen, Victoria Maureen F., Zhang Fuping, Eussen Bert E., van Ommen Gert-Jan B., Blonden Lau A.J., Riggins Gregory J., Chastain Jane L., Kunst Catherine B., Galjaard Hans, Thomas Caskey C., Nelson David L., Oostra Ben A., Warren Stephen T. (1991). Identification of a gene (FMR-1) containing a CGG repeat coincident with a breakpoint cluster region exhibiting length variation in fragile X syndrome. Cell.

[3] Turner G. (1997). Fragile X Syndrome: Diagnosis, Treatment and Research. Journal of Medical Genetics.

[4] Darnell Jennifer C., Jensen Kirk B., Jin Peng, Brown Victoria, Warren Stephen T., Darnell Robert B. (2001). Fragile X Mental Retardation Protein Targets G Quartet mRNAs Important for Neuronal Function. Cell.

[5] Darnell Jennifer C., Van Driesche Sarah J., Zhang Chaolin, Hung Ka Ying Sharon, Mele Aldo, Fraser Claire E., Stone Elizabeth F., Chen Cynthia, Fak John J., Chi Sung Wook, Licatalosi Donny D., Richter Joel D., Darnell Robert B. (2011). FMRP Stalls Ribosomal Translocation on mRNAs Linked to Synaptic Function and Autism. Cell.

[6] Qin Mei, Schmidt Kathleen C, Zametkin Alan J, Bishu Shrinivas, Horowitz Lisa M, Burlin Thomas V, Xia Zengyan, Huang Tianjiang, Quezado Zenaide M, Smith Carolyn Beebe (2013). Altered Cerebral Protein Synthesis in Fragile X Syndrome: Studies in Human Subjects and Knockout Mice. Journal of Cerebral Blood Flow & Metabolism.

[7] Qin M. (2005). Postadolescent Changes in Regional Cerebral Protein Synthesis: An In Vivo Study in the Fmr1 Null Mouse. Journal of Neuroscience.

[8] Osterweil Emily K., Chuang Shih-Chieh, Chubykin Alexander A., Sidorov Michael, Bianchi Riccardo, Wong Robert K.S., Bear Mark F. (2013). Lovastatin Corrects Excess Protein Synthesis and Prevents Epileptogenesis in a Mouse Model of Fragile X Syndrome. Neuron.

[9] Sharma A., Hoeffer C. A., Takayasu Y., Miyawaki T., McBride S. M., Klann E., Zukin R. S. (2010). Dysregulation of mTOR Signaling in Fragile X Syndrome. Journal of Neuroscience.

[10] Dölen Gül, Osterweil Emily, Rao B.S. Shankaranarayana, Smith Gordon B., Auerbach Benjamin D., Chattarji Sumantra, Bear Mark F. (2007). Correction of Fragile X Syndrome in Mice. Neuron.

[11] Henderson C., Wijetunge L., Kinoshita M. N., Shumway M., Hammond R. S., Postma F. R., Brynczka C., Rush R., Thomas A., Paylor R., Warren S. T., Vanderklish P. W., Kind P. C., Carpenter R. L., Bear M. F., Healy A. M. (2012). Reversal of Disease-Related Pathologies in the Fragile X Mouse Model by Selective Activation of GABAB Receptors with Arbaclofen. Science Translational Medicine.

[12] Bhattacharya Aditi, Kaphzan Hanoch, Alvarez-Dieppa Amanda C., Murphy Jaclyn P., Pierre Philippe, Klann Eric (2012). Genetic Removal of p70 S6 Kinase 1 Corrects Molecular, Synaptic, and Behavioral Phenotypes in Fragile X Syndrome Mice. Neuron.

[13] Michalon Aubin, Sidorov Michael, Ballard Theresa M., Ozmen Laurence, Spooren Will, Wettstein Joseph G., Jaeschke Georg, Bear Mark F., Lindemann Lothar (2012). Chronic Pharmacological mGlu5 Inhibition Corrects Fragile X in Adult Mice. Neuron.

[14] Jacquemont Sébastien, Pacini Laura, Jønch Aia E, Cencelli Giulia, Rozenberg Izabela, He Yunsheng, D’Andrea Laura, Pedini Giorgia, Eldeeb Marwa, Willemsen Rob, Gasparini Fabrizio, Tassone Flora, Hagerman Randi, Gomez-Mancilla Baltazar, Bagni Claudia (2018). Protein synthesis levels are increased in a subset of individuals with fragile X syndrome. Human Molecular Genetics.

[15] Gross Christina, Bassell Gary J. (2011). Excess Protein Synthesis in FXS Patient Lymphoblastoid Cells Can Be Rescued with a p110β-Selective Inhibitor. Molecular Medicine.

[16] Kumari Daman, Bhattacharya Aditi, Nadel Jeffrey, Moulton Kristen, Zeak Nicole M., Glicksman Anne, Dobkin Carl, Brick David J., Schwartz Philip H., Smith Carolyn B., Klann Eric, Usdin Karen (2014). Identification of Fragile X Syndrome Specific Molecular Markers in Human Fibroblasts: A Useful Model to Test the Efficacy of Therapeutic Drugs. Human Mutation.

[17] Utami KH, et al. Integrative analysis identifies key molecular signatures underlying neurodevelopmental deficits in fragile X syndrome. Biol Psych. 2020. 10.1016/j.biopsych.2020.05.005. 10.1016/j.biopsych.2020.05.005 32653109

[18] Achuta VS, et al. Metabotropic glutamate receptor 5 responses dictate differentiation of neural progenitors to NMDA-responsive cells in fragile X syndrome. Dev Neurobiol. 2016. 10.1002/dneu.22419 27411166

[19] Li W., Sun W., Zhang Y., Wei W., Ambasudhan R., Xia P., Talantova M., Lin T., Kim J., Wang X., Kim W. R., Lipton S. A., Zhang K., Ding S. (2011). Rapid induction and long-term self-renewal of primitive neural precursors from human embryonic stem cells by small molecule inhibitors. Proceedings of the National Academy of Sciences.

[20] Schmidt Enrico K, Clavarino Giovanna, Ceppi Maurizio, Pierre Philippe (2009). SUnSET, a nonradioactive method to monitor protein synthesis. Nature Methods.

[21] Osterweil E. K., Krueger D. D., Reinhold K., Bear M. F. (2010). Hypersensitivity to mGluR5 and ERK1/2 Leads to Excessive Protein Synthesis in the Hippocampus of a Mouse Model of Fragile X Syndrome. Journal of Neuroscience.

[22] Brennand Kristen J., Simone Anthony, Jou Jessica, Gelboin-Burkhart Chelsea, Tran Ngoc, Sangar Sarah, Li Yan, Mu Yangling, Chen Gong, Yu Diana, McCarthy Shane, Sebat Jonathan, Gage Fred H. (2011). Modelling schizophrenia using human induced pluripotent stem cells. Nature.

[23] Gantois Ilse, Khoutorsky Arkady, Popic Jelena, Aguilar-Valles Argel, Freemantle Erika, Cao Ruifeng, Sharma Vijendra, Pooters Tine, Nagpal Anmol, Skalecka Agnieszka, Truong Vinh T, Wiebe Shane, Groves Isabelle A, Jafarnejad Seyed Mehdi, Chapat Clément, McCullagh Elizabeth A, Gamache Karine, Nader Karim, Lacaille Jean-Claude, Gkogkas Christos G, Sonenberg Nahum (2017). Metformin ameliorates core deficits in a mouse model of fragile X syndrome. Nature Medicine.

[24] Howell Jessica J., Hellberg Kristina, Turner Marc, Talbott George, Kolar Matthew J., Ross Debbie S., Hoxhaj Gerta, Saghatelian Alan, Shaw Reuben J., Manning Brendan D. (2017). Metformin Inhibits Hepatic mTORC1 Signaling via Dose-Dependent Mechanisms Involving AMPK and the TSC Complex. Cell Metabolism.

[25] Gillespie ZE, et al. Metformin induces the AP-1 transcription factor network in normal dermal fibroblasts. Sci Rep. 2019. 10.1038/s41598-019-41839-1. 10.1038/s41598-019-41839-1 PMC6441003 30926854

[26] Castren M., Tervonen T., Karkkainen V., Heinonen S., Castren E., Larsson K., Bakker C. E., Oostra B. A., Akerman K. (2005). Altered differentiation of neural stem cells in fragile X syndrome. Proceedings of the National Academy of Sciences.

[27] Luo Yuping, Shan Ge, Guo Weixiang, Smrt Richard D., Johnson Eric B., Li Xuekun, Pfeiffer Rebecca L., Szulwach Keith E., Duan Ranhui, Barkho Basam Z., Li Wendi, Liu Changmei, Jin Peng, Zhao Xinyu (2010). Fragile X Mental Retardation Protein Regulates Proliferation and Differentiation of Adult Neural Stem/Progenitor Cells. PLoS Genetics.

[28] Callan M. A., Cabernard C., Heck J., Luois S., Doe C. Q., Zarnescu D. C. (2010). Fragile X protein controls neural stem cell proliferation in the Drosophila brain. Human Molecular Genetics.

[29] Xiong ZS, et al. Effect of metformin on cell proliferation, apoptosis, migration and invasion in A172 glioma cells and its mechanisms. Mol Med Rep. 2019. 10.3892/mmr.2019.10369. 10.3892/mmr.2019.10369 PMC6625203 31173255

[30] Liang Xue, Kong Peiyan, Wang Jin, Xu Yulin, Gao Chunfang, Guo Guozhen (2017). Effects of metformin on proliferation and apoptosis of human megakaryoblastic Dami and MEG-01 cells. Journal of Pharmacological Sciences.

[31] Xie Wei, Wang Lei, Sheng Halei, Qiu Jing, Zhang Di, Zhang Le, Yang Fan, Tang Dahai, Zhang Kebin (2017). Metformin Induces Growth Inhibition and Cell Cycle Arrest by Upregulating MicroRNA34a in Renal Cancer Cells. Medical Science Monitor.

[32] Sheridan Steven D., Theriault Kraig M., Reis Surya A., Zhou Fen, Madison Jon M., Daheron Laurence, Loring Jeanne F., Haggarty Stephen J. (2011). Epigenetic Characterization of the FMR1 Gene and Aberrant Neurodevelopment in Human Induced Pluripotent Stem Cell Models of Fragile X Syndrome. PLoS ONE.

[33] M., T., L., K.-Y., M., S. & Ben-Yosef D. AO - Telias Liron; ORCID: http://orcid.org/0000-0002-9705-6697, M. O. http://orcid.org/000-0002-7632-6942 A. O.-K.-Y. Functional deficiencies in fragile X neurons derived from human embryonic stem cells. J Neurosci (2015). 10.1523/JNEUROSCI.0317-15.2015 PMC6605488 26586818

[34] Doers ME, et al. IPSC-derived forebrain neurons from FXS individuals show defects in initial neurite outgrowth. Stem Cells Dev. 2014. 10.1089/scd.2014.0030 PMC4103262 24654675

[35] Richter Joel D., Bassell Gary J., Klann Eric (2015). Dysregulation and restoration of translational homeostasis in fragile X syndrome. Nature Reviews Neuroscience.

[36] Berry-Kravis Elizabeth (2014). Mechanism-Based Treatments in Neurodevelopmental Disorders: Fragile X Syndrome. Pediatric Neurology.

[37] Lee Anna, Ventola Pamela, Budimirovic Dejan, Berry-Kravis Elizabeth, Visootsak Jeannie (2018). Clinical Development of Targeted Fragile X Syndrome Treatments: An Industry Perspective. Brain Sciences.

[38] Erickson CA, et al. Fragile X targeted pharmacotherapy: lessons learned and future directions. J Neurodev Disord. 2017. 10.1186/s11689-017-9186-9. 10.1186/s11689-017-9186-9 PMC5467059 28616096

[39] Jeste Shafali S., Geschwind Daniel H. (2016). Clinical trials for neurodevelopmental disorders: At a therapeutic frontier. Science Translational Medicine.

[40] Wang X, et al. Activation of the ERK pathway contributes to the behavioral deficit of fragile X-syndrome. J Neurochem. 2012. 10.1111/j.1471-4159.2012.07722.x 22393900

[41] Monyak R E, Emerson D, Schoenfeld B P, Zheng X, Chambers D B, Rosenfelt C, Langer S, Hinchey P, Choi C H, McDonald T V, Bolduc F V, Sehgal A, McBride S M J, Jongens T A (2016). Insulin signaling misregulation underlies circadian and cognitive deficits in a Drosophila fragile X model. Molecular Psychiatry.

[42] Dy A.B.C., Tassone F., Eldeeb M., Salcedo-Arellano M.J., Tartaglia N., Hagerman R. (2017). Metformin as targeted treatment in fragile X syndrome. Clinical Genetics.

[43] Protic D, et al. Cognitive and behavioral improvement in adults with fragile X syndrome treated with metformin-two cases. Mol Genet Genomic Med. 2019. 10.1002/mgg3.745. 10.1002/mgg3.745 PMC6625129 31104364

[44] Gantois Ilse, Popic Jelena, Khoutorsky Arkady, Sonenberg Nahum (2019). Metformin for Treatment of Fragile X Syndrome and Other Neurological Disorders. Annual Review of Medicine.

