# Supplementary material for: Elevated de novo protein synthesis in FMRP-deficient human neurons and its correction by metformin treatment
Source: Mol Autism. 2020 May 27;11:41. doi: 10.1186/s13229-020-00350-5 (PMC7251671; doi:10.1186/s13229-020-00350-5)
Supplement: Supplementary file 1 — Additional file 1: Figure S1. Assessment of FMRP expression in control and FMR1KO ESC, NPC and neurons. The expression of FMRP was assessed in ESC, NPCs and neurons by immunoblot. FMR1KO showed absence of FMRP expression in all cell types. CON = control; KO = FMR1KO. Figure S2. Assessment of de novo protein synthesis and phosphorylation of ERK1/2 and Akt in FXS NPCs treated with 0.5 mM metformin. (A) Protein synthesis (SUnSET assay) was performed in NPCs treated with 0.5 mM metformin. A representative immunoblot image is shown with 2 replicates per group. Relative expression of puromycin was quantified by ImageJ and normalized to control-vehicle. Values shown as mean ± SEM (n=4 per group). *p < 0.05 and **p < 0.01 by one-way ANOVA with Fisher’s LSD post-hoc test. (B) Levels of phosphorylated Akt, total Akt, phosphorylated ERK and total ERK in untreated and metformin-treated NPCs were assessed by immunoblot. Values shown as mean ± SEM (n=3 per group). *p < 0.05 by one-way ANOVA with Fisher’s LSD post-hoc test. Figure S3. Assessment of de novo protein synthesis and phosphorylation of ERK1/2 and Akt in FXS NPCs treated with 0.1 and 1 mM metformin (A) Protein synthesis (SUnSET assay) was performed in NPCs treated with 0.1 and 1 mM metformin. A representative immunoblot image is shown. Relative expression of puromycin was quantified by ImageJ and normalized to the control-vehicle. Values shown as mean ± SEM from three replicates per genotype, from two immunoblot experiments. * p < 0.05 as determined by one-way ANOVA with Tukey post-hoc test. (B) Expression of phosphorylated Akt, total Akt, phosphorylated ERK and total ERK for untreated and metformin-treated condition was assessed by immunoblotting. A representative immunoblot image is shown. Values shown as mean ± SEM (n=4 per group). *p < 0.05, as determined by two-way ANOVA with Fisher’s LSD post-hoc test. Figure S4. No effect of metformin treatment on control or FMR1KO NPC counts. Total number of control and FMR1KO ce [file 13229_2020_350_MOESM1_ESM.docx]

**Supplementary Figures**

**Elevated *de novo* protein synthesis in FMRP-deficient human neurons and its correction by metformin treatment**


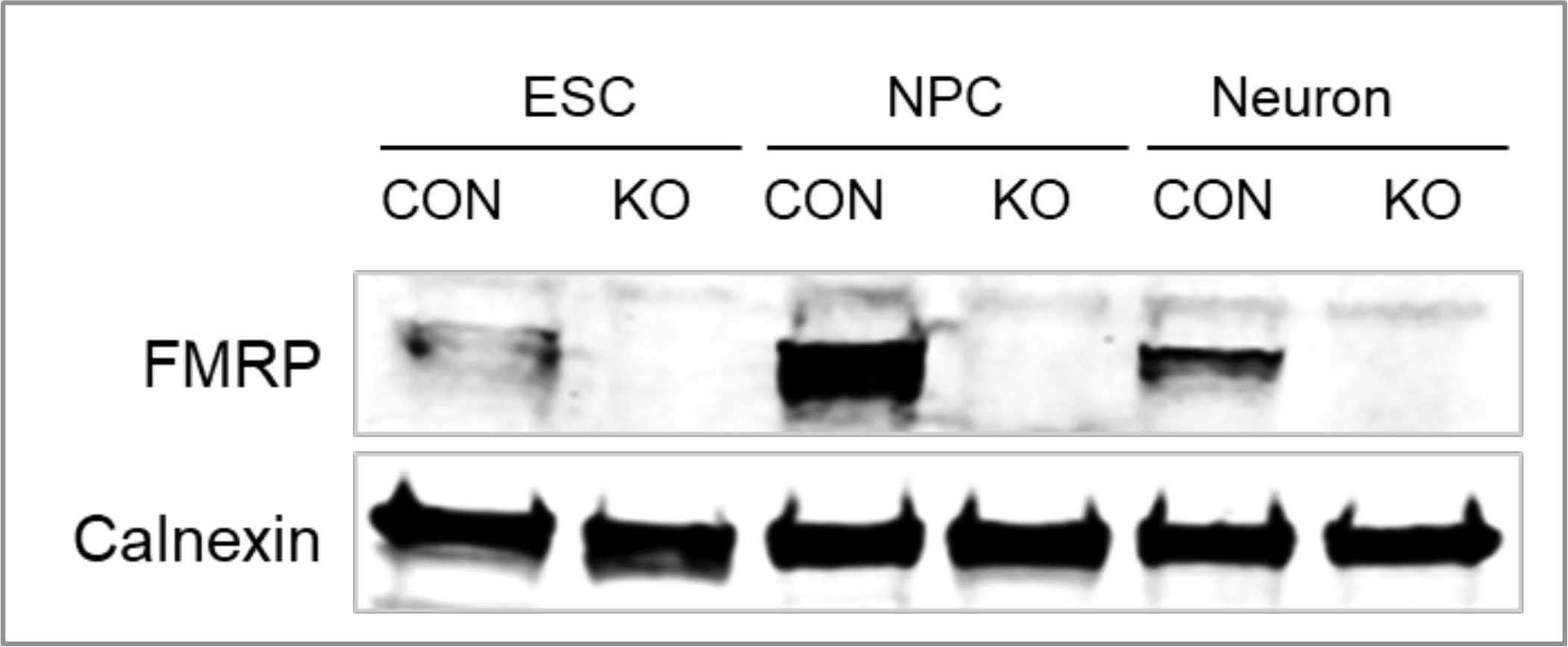


**Figure S1. Assessment of FMRP expression in control and *FMR1*KO ESC, NPC and neurons.** The expression of FMRP was assessed in ESC, NPCs and neurons by immunoblot. *FMR1*KO showed absence of FMRP expression in all cell types. CON = control; KO = *FMR1*KO.


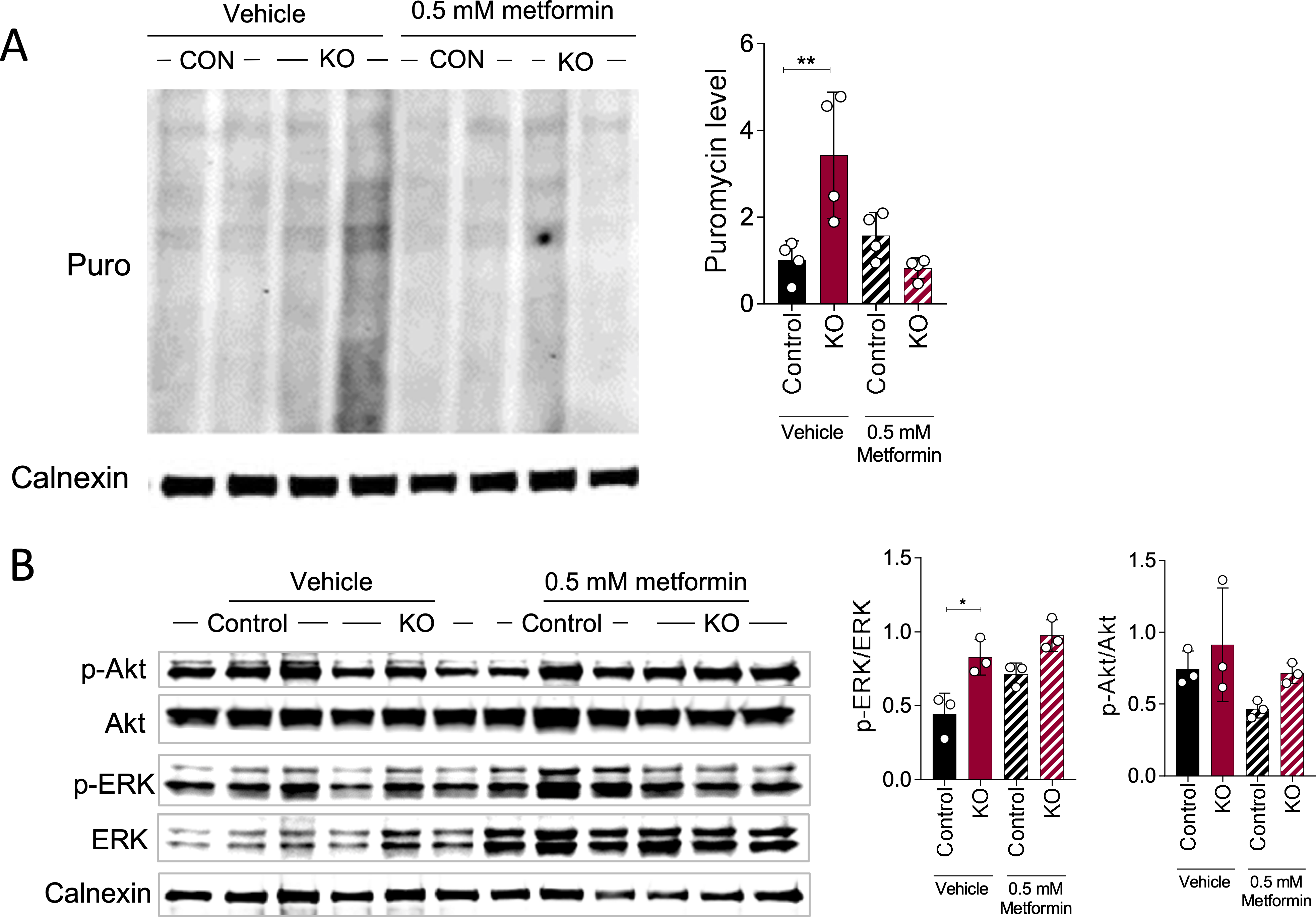


**Figure S2. Assessment of *de novo* protein synthesis and phosphorylation of ERK1/2 and Akt in FXS NPCs treated with 0.5 mM metformin.**

(A) Protein synthesis (SUnSET assay) was performed in NPCs treated with 0.5 mM metformin. A representative immunoblot image is shown with 2 replicates per group. Relative expression of puromycin was quantified by ImageJ and normalized to control-vehicle. Values shown as mean ± SEM (n=4 per group). *p < 0.05 and **p < 0.01 by one-way ANOVA with Fisher’s LSD post-hoc test. (B) Levels of phosphorylated Akt, total Akt, phosphorylated ERK and total ERK in untreated and metformin-treated NPCs were assessed by immunoblot. Values shown as mean ± SEM (n=3 per group). *p < 0.05 by one-way ANOVA with Fisher’s LSD post-hoc test.


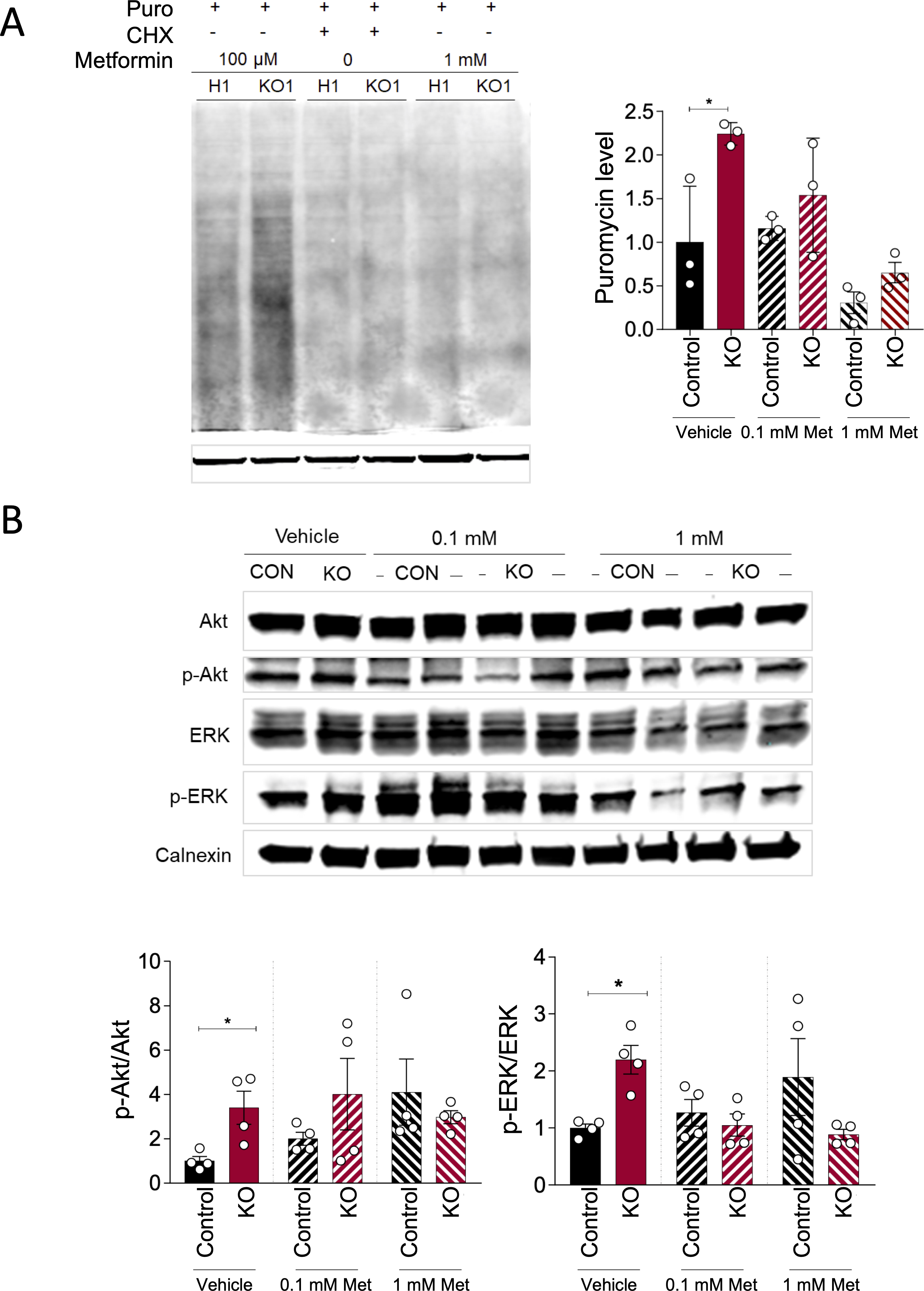


**Figure S3. Assessment of *de novo* protein synthesis and phosphorylation of ERK1/2 and Akt in FXS NPCs treated with 0.1 and 1 mM metformin**

(A) Protein synthesis (SUnSET assay) was performed in NPCs treated with 0.1 and 1 mM metformin. A representative immunoblot image is shown. Relative expression of puromycin was quantified by ImageJ and normalized to the control-vehicle. Values shown as mean ± SEM from three replicates per genotype, from two immunoblot experiments. * p < 0.05 as determined by one-way ANOVA with Tukey post-hoc test. (B) Expression of phosphorylated Akt, total Akt, phosphorylated ERK and total ERK for untreated and metformin-treated condition was assessed by immunoblotting. A representative immunoblot image is shown. Values shown as mean ± SEM (n=4 per group). *p < 0.05, as determined by two-way ANOVA with Fisher’s LSD post-hoc test.


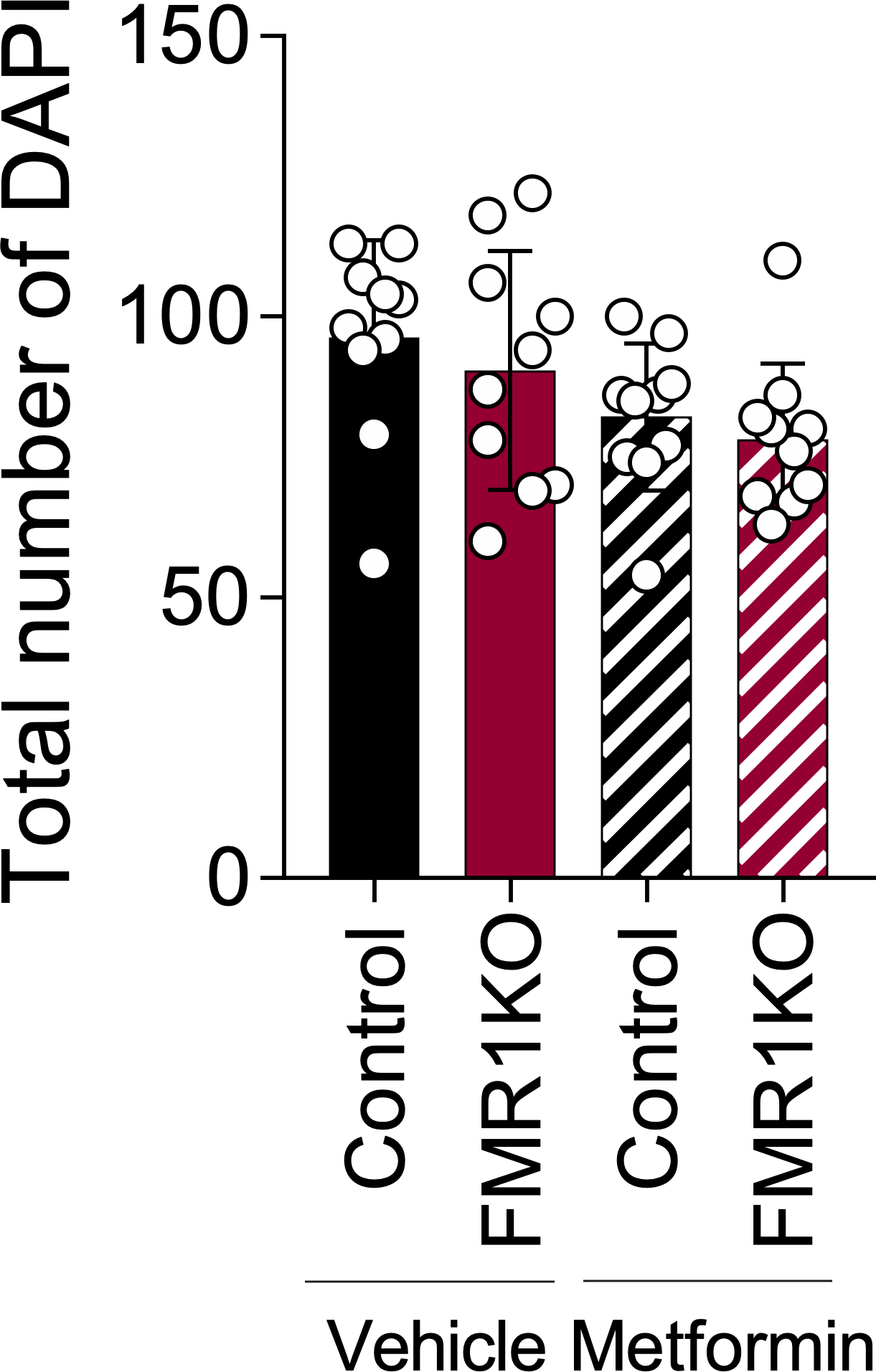


**Figure S4. No effect of metformin treatment on control or *FMR1*KO NPC counts.**

Total number of control and *FMR1*KO cells (based on DAPI-positive cells) in NPCs cultures treated with vehicle or metformin (1 mM). There were no significant differences among the groups and treatment conditions. Values shown are based on 10 image fields per group and treatment condition.


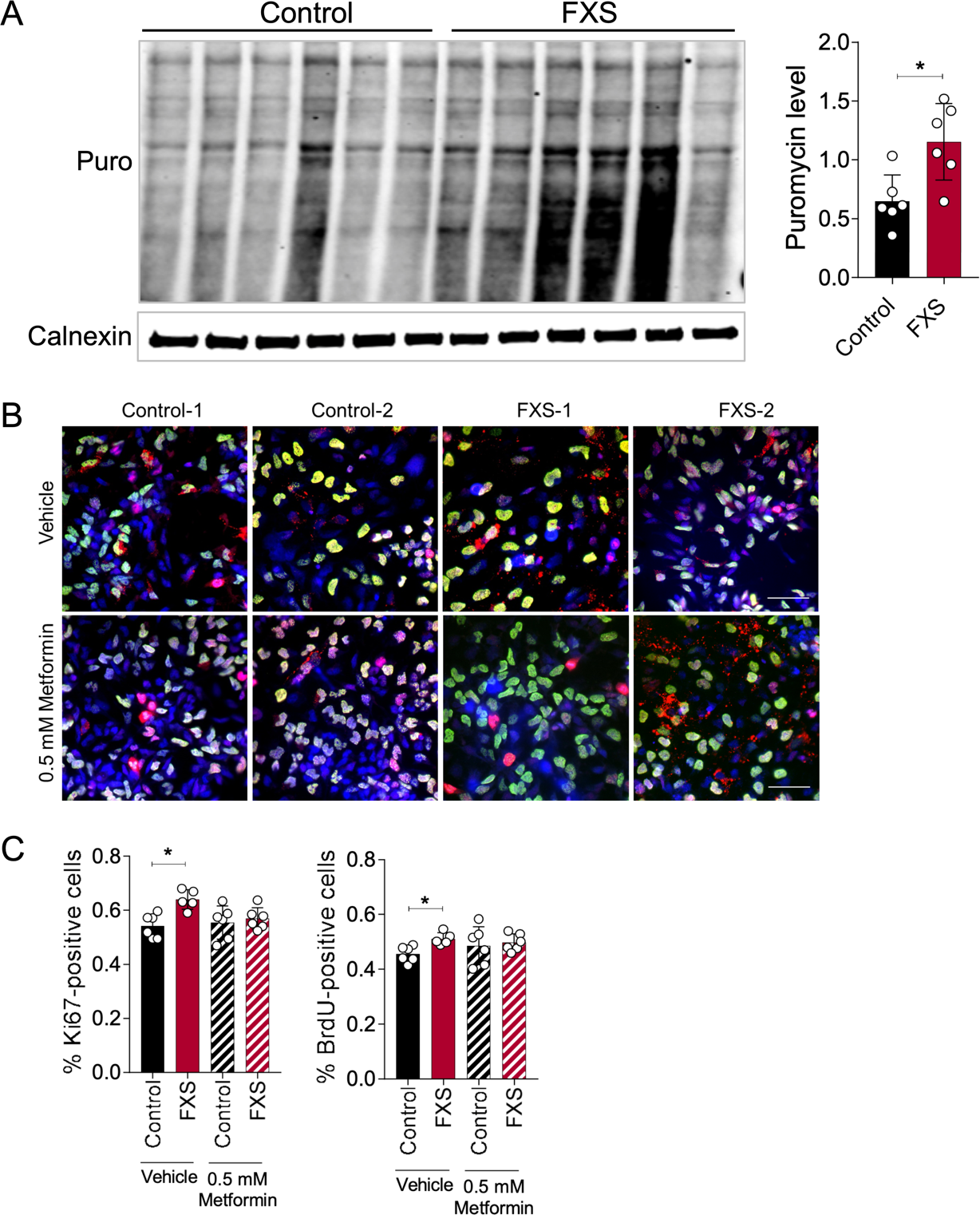


**Figure S5. Assessment of *de novo* protein synthesis and metformin effect on proliferation in FXS and control hiPSC-derived NPCs.** (**a**) Protein synthesis (SUnSET assay) was performed in 2 Control and 2 FXS hiPSC-derived NPCs. Relative expression of puromycin was quantified by ImageJ. Values shown as mean ± SEM from three replicates per genotype. *p < 0.05 and **p < 0.01 by one-way ANOVA with Fisher LSD post-hoc test; (**b**) Immunostaining shows proliferative markers BrdU (Green) and Ki67 (Red) expression. BrdU-labelling and Ki67 reveals increased proliferation in FXS iPSC-derived NPCs compared to control in the vehicle-treated condition. Treatment with 0.5 mM metformin ameliorates the excessive proliferation rate in the FXS hiPSC-derived NPCs. Scale bar = 50 µm; (**c**) Quantification of BrdU- and Ki67-positive cells by ImageJ. Values shown as mean ± SEM based on blinded counting of 8 images from three coverslips per cell line.
